# A guide to bayesian networks software for structure and parameter learning, with a focus on causal discovery tools

**DOI:** 10.3389/fsysb.2025.1631901

**Published:** 2025-08-25

**Authors:** Francesco Canonaco, Joverlyn Gaudillo, Nicole Astrologo, Fabio Stella, Enzo Acerbi

**Affiliations:** ^1^ Minutia.AI Pte. Ltd., Singapore, Singapore; ^2^ Department of Informatics, Systems and Communication, University of Milano-Bicocca, Milano, Italy

**Keywords:** structure learning, parameter learning, causal discovery algorithms, causal discovery, bayesian networks (BNs)

## Abstract

A representation of the cause-effect mechanism is needed to enable artificial intelligence to represent how the world works. Bayesian Networks (BNs) have proven to be an effective and versatile tool for this task. BNs require constructing a structure of dependencies among variables and learning the parameters that govern these relationships. These tasks, referred to as structural learning and parameter learning, are actively investigated by the research community, with several algorithms proposed and no single method having established itself as standard. A wide range of software, tools, and packages have been developed for BNs analysis and made available to academic researchers and industry practitioners. As a consequence of having no one-size-fits-all solution, moving the first practical steps and getting oriented into this field is proving to be challenging to outsiders and beginners. In this paper, we review the most relevant tools and software for BNs structural and parameter learning to date, with a focus on causal discovery tools, providing our subjective recommendations directed to an audience of beginners. In addition, we provide an extensive easy-to-consult overview table summarizing all software packages and their main features. By improving the reader’s understanding of which available software might best suit their needs, we improve accessibility to the field and make it easier for beginners to take their first step into it.

## 1 Introduction

**FIGURE 1:**
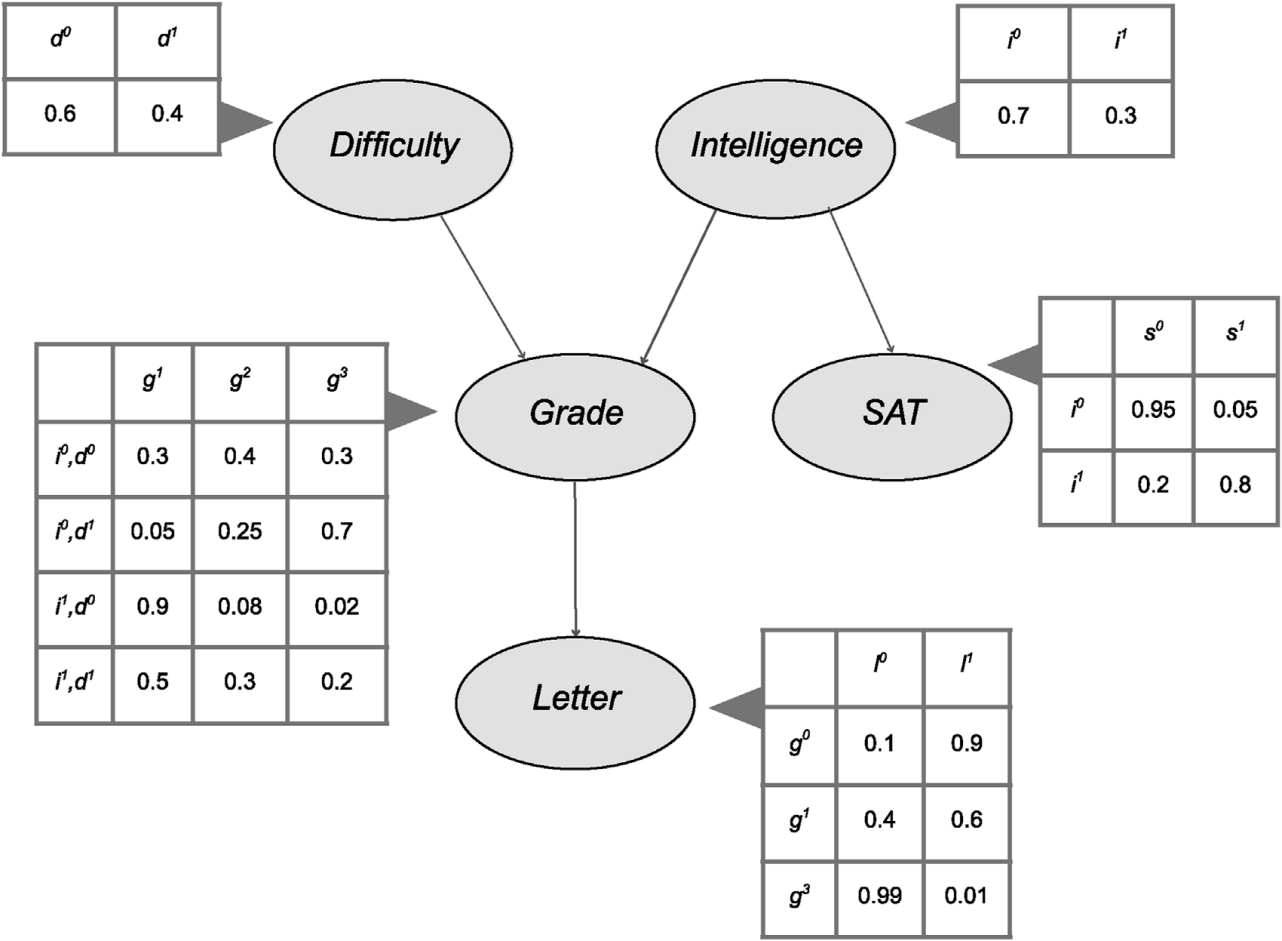
Student Bayesian Network example with CPDs.

Bayesian networks (BNs) have established themselves over the years as a powerful framework for modeling and analyzing complex systems under conditions of uncertainty. They have been widely employed in fields such as medicine (Arora et al., 2019), biology (Needham et al., 2007) and engineering (Kammouh et al., 2020). BNs represent probabilistic relationships among variables in a graphical way that allows efficient inference and intuitive causal reasoning when specific assumptions are met. It is important to clarify that while Bayesian networks encode conditional dependencies through directed edges, these do not necessarily imply causal relationships. A causal network is a specific type of Bayesian network where the edges reflect actual causal influences among variables, and their interpretation relies on assumptions such as causal sufficiency, faithfulness, and the absence of unmeasured confounding. Throughout this paper, we include structure learning algorithms developed for both probabilistic modeling and causal discovery. For a detailed discussion of the assumptions underlying causal discovery, we refer the reader to (Vonk et al., 2023). A BN (Jensen and Nielsen, 2007) consists of:

• A collection of random variables represented as nodes $X=\left\{X_{1},X_{2},\ldots ,X_{n}\right\}$ , connected by directed edges that form a Directed Acyclic Graph (DAG). For instance, in Figure 1, the variables could be denoted as D (Difficulty), I (Intelligence), G (Grade), S (SAT), and L (Letter), corresponding to the nodes shown in the DAG.

• A finite set of mutually exclusive states associated with each random variable.

• For each random variable $X_{i}$ with parents $\text{Pa}\left(X_{i}\right)=\left\{Y_{1},\ldots ,Y_{n}\right\}$ , a Conditional Probability Distribution (CPD) specifying the probability distribution $P\left(X_{i}|Y_{1},\ldots ,Y_{n}\right)$ . This CPD quantifies the influence of the parent variables on $X_{i}$ . If $X_{i}$ has no parents, it is associated with an unconditional probability distribution $P\left(X_{i}\right)$ . In Figure 1, Pa(G)={D,I} , which means that G depends on both D and I via the conditional distribution P(G∣D,I) .

Figure 1 shows a well-known example of BN where the variables course *Difficulty* and student *Intelligence* are assumed to be independently assigned prior to the realization (observing the value) of any other variable. The student’s *Grade* is influenced by both *Difficulty* and *Intelligence*. The *SAT* Score depends solely on *Intelligence*, while the recommendation *Letter* is assumed to be based exclusively on the *Grade*. This structure reflects the intuitive idea that each variable is directly influenced only by its parent nodes in the network (Koller and Friedman, 2009).

In fact, BNs leverage conditional independence to compactly represent the joint probability distribution over a set of random variables $X=\left\{X_{1},X_{2},\ldots ,X_{n}\right\}$ . The joint distribution can be factorized into a product of CPDs, one for each node:

$$P\left(X_{1},X_{2},\ldots ,X_{n}\right)=\overset{n}{\underset{i=1}{\prod }}P\left(X_{i}|\text{Pa}\left(X_{i}\right)\right)$$

where $\text{Pa}\left(X_{i}\right)$ denotes the set of parent variables of $X_{i}$ in the network. Although, in principle, various types of distributions can be used, most applications in the literature have focused on two main modeling assumptions due to their mathematical tractability and computational efficiency:

• **Discrete Bayesian Networks** (Heckerman et al., 1995): assume that $X_{i}$ is a multinomial random variable dependent on the configurations of the values of its parents;

• **Gaussian Bayesian Networks** (Geiger and Heckerman, 1994): assume that each variable $X_{i}$ is a univariate normal random variable, with its value linearly dependent on its parent variables.

The objective of the learning process is to determine both the network structure and the associated parameters that best represent the observed data. Learning a Bayesian Network involves:

• **Structure learning**: identifying the qualitative structure of the network, i.e., the conditional independence relationships among the variables.

• **Parameter learning**: estimating the conditional probability distributions (CPDs) for each node.

Learning the structure of a BN from data is a foundational step of the model construction process. For this purpose, a multitude of algorithms have been developed over the years; these methods are typically categorized into three groups: *constraint-based*, *score-based*, and *hybrid*.

*Constraint-based* algorithms rely on the theory of causal graphical models introduced by Pearl (Verma and Pearl, 1990). A well-known example of this class is the PC-Stable (named after its authors Peter and Clark) algorithm (Colombo and Maathuis, 2014), which improves the original PC algorithm (Spirtes et al., 2000) by making it more robust to variable ordering. The algorithm starts with a complete undirected graph and recursively removes edges using a conditional independence (CI) test. *Score-based* algorithms define a scoring function, such as BIC (Bayesian information criterion) (Neath and Cavanaugh, 2012), AIC (Akaike information criterion) (Cavanaugh and Neath, 2019), to evaluate how well a given network fits the data. A search algorithm, such as greedy search or simulated annealing, is then used to explore the space of possible graphs. *Hybrid* algorithms combine constraint-based and score-based approaches. Typically, a constraint-based method is used to reduce the search space, followed by a score-based optimization over the reduced space.

These algorithms generally assume that the input is *tabular data*, where each row represents an independent observation (i.i.d.), and each column corresponds to a variable. *Constraint-based* methods require data that are suitable for conditional independence (CI) testing, which typically includes discrete or continuous variables depending on the CI test used (e.g., chi-square for discrete, partial correlation for continuous). *Score-based* methods, on the other hand, rely on likelihood-based scoring functions and can handle discrete, continuous, or mixed data types depending on the scoring function and underlying assumptions. Hybrid methods inherit the data requirements of both approaches.

To speed up or improve structure learning, prior knowledge can be incorporated to constrain or guide the search for the network structure. Users may specify relationships that are known to exist, permitted, or prohibited, thereby reducing the search space and enhancing both the accuracy and efficiency of learning algorithms.

An overview of structure learning approaches is beyond the scope of this document; a comprehensive assessment of state-of-the-art methodologies can be found in (Nogueira et al., 2022; Kitson et al., 2023; Glymour et al., 2019; Scanagatta et al., 2019). Moreover, readers interested in the performance of the different classes of algorithms can refer to dedicated publications that offer comprehensive evaluations of the accuracy and computational efficiency of structure learning methods (Scutari et al., 2019).

Parameter learning is another critical task in BNs development. Given the DAG, the objective of parameter learning is to estimate the parameters of the conditional probability distributions associated with each node, which is essential for inference and prediction. For a comprehensive review of parameter learning strategies, challenges, and algorithms, refer to the works of (Ji et al., 2015; Heckerman, 1998).

Approaching the study of BN framework requires a solid understanding of fundamental principles in disciplines such as probability and computer science. Assuming that the reader is already familiar with these foundations, some convenient readings on causality and BNs science are offered by Probabilistic Graphical Models Principles and Techniques (Koller and Friedman, 2009), Bayesian Artificial Intelligence (Korb and Nicholson, 2010), Probabilistic Reasoning in Intelligent Systems (Pearl, 2014), Bayesian Networks with Examples in R (Scutari and Denis, 2021), Bayesian Networks in R with Application in the field of System Biology (Scutari and Lebre, 2013), Bayesian Networks and Influence Diagrams (Kjaerulff and Madsen, 2008). This document assumes that the reader is equipped with the necessary foundational knowledge and is ready to engage in practical hands-on work.

Over the past 5 years, the field of causality and BNs development has seen an influx of numerous packages with no single solution being able to cater to all requirements and scenarios; this abundance of options is often challenging for individuals trying to gain hands-on experience with BNs. This document simplifies structure and parameter learning in BNs by providing a comprehensive overview of available software packages with a focus on causal discovery. In addition, we offer our subjective recommendations on selecting the best tools based on the reader’s specific objectives. The remainder of this paper is structured as follows: Section 2 provides a systematic review of both open-source and commercial software. Section 3 offers guidance on selecting tools suitable for beginners. Section 4 summarizes the key contributions of this work. A concise summary of all reviewed tools is provided in Supplementary Table S1 (Supplementary Material).

## 2 Software tools and packages

### 2.1 gCastle

gCastle (Zhang et al., 2021) is an end-to-end Python toolbox created by Huawei Noah’s Ark Lab for causal structure learning. The package is equipped with functionalities such as data generation from simulated or real-world datasets, causal structure learning, and evaluation metrics.

### 2.2 bnlearn

bnlearn (Scutari, 2010) is an R package developed by Marco Scutari and first released in 2007 with functionality to learn the structure of BNs, parameter estimation, and inference. After 10 years of continuous development, the package has grown to accommodate a multitude of algorithms from the literature. The package implements constraint-based algorithms, e.g., Peter-Clark (PC), Grow-Shrink (GS), Incremental Association Markov Blanket (IAMB), Inter-IAMB, Fast-IAMB, IAM-False Discovery Rate (FDR), Semi-Interleaved HITON-PC, and Max-Min Parents and Children (MMPC), pairwise-based algorithms, e.g., Algorithm for the Reconstruction of Accurate Cellular Networks (ARACNe) and Chow-Liu (ARACNE and Chow-Liu), score-based, e.g., Hill-Climbing (HC) and Tabu Search, hybrid algorithms, e.g., Hybrid Parents and Children (HPC), Max-Min HC (MMHC), Restricted Structural Maximum Algorithm 2 (RSMAX2), and Tree-augmented Naive Bayes (TAN), structure learning algorithms for discrete, Gaussian and conditional Gaussian networks, along with many score functions and conditional independence tests. Some utility functions (model comparison and manipulation, random data generation, arc orientation testing, simple and advanced plots) are included, as well as support for parameter estimation, e.g., maximum likelihood estimation (MLE) and Bayesian estimation, and inference, conditional probability queries, cross-validation, bootstrap, and model averaging.

### 2.3 pgmpy

Pgmpy (Ankan and Panda, 2015) is a Python library developed in 2015 by Ankur Ankan to work with probabilistic graphical models. It allows users to create their graphical models and then perform inferences or map queries to them. The library implements several inference algorithms like variable elimination, belief propagation, etc. The library is designed with a modular structure, allowing users to access dedicated classes for commonly used graphical models like Naive Bayes (NB) and hidden Markov models, eliminating the need to build them from base models. Currently, it includes implementations of various algorithms for structure learning, parameter estimation, both approximate, i.e., sampling-based, and exact inference, as well as causal inference.

### 2.4 Tetrad

Tetrad (Ramsey et al., 2018) is a Java suite of software for the discovery, estimation, and simulation of causal models developed by the Carnegie Mellon University-Causal Learning and Reasoning (CMU-CLeaR) group. Some of its basic features for beginners include the ability to load existing datasets, load existing causal graphs, and create a new causal graph. For practitioners, the tool is equipped with advanced functionalities, such as specifying prior knowledge on constraint-based algorithms, manipulating data by imputing missing values, discretizing data, simulating data from statistical models, and computing the probability distribution of any variable, among others. It features a graphical user interface (GUI) and offers popular constraint-based algorithms for causal discovery such as PC, Fast Causal Inference (FCI), PC-Max, Conservative PC (CPC), and MLE for parameter learning.

### 2.5 Causal command (CMD)

Causal-cmd^1^ is a Java application that offers a command-line interface tool for causal discovery algorithms developed by the Center for Causal Discovery. Currently, the application includes more than 30 algorithms for causal discovery.

### 2.6 Causal-learn

Causal-learn (Zheng et al., 2024) is a Python translation and extension of the Tetrad Java code (refer to the Tetrad package) developed by CMU-CLeaR group. It offers implementations of up-to-date causal discovery methods, as well as simple and intuitive Application Programming Interfaces (APIs).

### 2.7 pcalg

Pcalg (Kalisch et al., 2012) is an R package developed by Markus Kalisch et al. in 2006. It offers constraint-based algorithms such as PC, FCI, and Really FCI (RFCI) as well as hybrid and score-based algorithms for causal discovery.

### 2.8 LiNGAM

Linear Non-Gaussian Acyclic Model (LiNGAM) (Shimizu et al., 2006) is a Python package for causal discovery developed by T. Ikeuchi et al. The package offers many causal discovery algorithms for linear non-Gaussian models such as Direct-LiNGAM, Linear Non-Gaussian Models for Latent Factors (LiNA), and Vector Autoregressive Models-LiNGAM (VAR-LiNGAM).

### 2.9 CDT

CDT (Kalainathan et al., 2020) is a Python package for causal inference in graphical models and pairwise settings (compatible with Python ≥ 3.5). Developed by Diviyan Kalainathan and Olivier Goudet, CDT provides tools for structure learning and dependency analysis. It leverages on NumPy, scikit-learn, PyTorch, and R to implement various algorithms for causal discovery, including methods from bnlearn and pcalg. The package is particularly suited for analyzing observational data, offering both classical and deep learning-based approaches to causal structure recovery.

### 2.10 pyAgrum

pyAgrum (Ducamp et al., 2020) is a Python wrapper for the C++ aGrUM library. It offers a high-level interface to aGrUM, enabling users to create, model, learn, apply, compute, and integrate BNs and other graphical models. Some specific (Python and C++) codes are added to simplify and extend the aGrUM API. The package contains causal discovery, parameter learning, and inference algorithms.

### 2.11 bnlearn (python)

Bnlearn^2^ is a Python package for causal discovery, parameter learning and inference developed by Erdogan Taskesen. It implements the most classical approaches for causal discovery such as HC, exhaustive search, Chow-Liu, TAN, PC, and MLE, as well as Bayesian estimation for parameter learning.

### 2.12 OpenMarkov

OpenMarkov (Arias et al., 2019) is a Java open-source software tool developed by the Research Centre for Intelligent Decision-Support Systems. OpenMarkov comes with a user interface and can perform causal discovery employing the PC algorithm and HC search.

### 2.13 pomegranate

Pomegranate (Schreiber, 2018), a Python package developed by Jacob Schreiber, offers efficient and versatile probabilistic models, spanning from individual probability distributions to composite models including BNs and hidden Markov models. The package offers both constraint-based and score-based algorithms, as well as parameter learning procedures.

### 2.14 BayesFusion

BayesFusion^3^ is a commercial software offering different solutions for causal discovery, parameter learning, and inference. Their flagship product is GeNIe, a tool for artificial intelligence and machine learning that has at its core the BN framework and other types of graphical probabilistic models. The SMILE engine allows the user to include custom applications that can be written in a variety of programming languages, e.g., C++, Python, Java, .NET, R, Matlab. Models created with GeNIe or SMILE can be shared or used on mobile devices via BayesMobile, or through a web browser with BayesBox.

### 2.15 BayesiaLab

BayesiaLab^4^ is a commercial software developed by Dr. Lionel Jouffe and Dr. Paul Munteanu and their team. It offers plenty of algorithms for causal discovery, parameter learning, and inference. The software includes a graphical user interface and is well documented.

### 2.16 Bayes Server

Bayes Server^5^ is a commercial software developed by Bayes Server Ltd. Besides the most well-known algorithms for causal discovery, parameter learning and inference, the software offers a wide range of tools for diagnostic, anomaly detection and decision-making under uncertainty which have at their core the BN framework. Bayes Server can be used in the cloud as well as on a local machine through a GUI. It offers an advanced user interface accessible programmatically via a number of APIs that can be used via Java, Matlab, Python, Spark and R.

## 3 My causal path: picking the right tool as a beginner

This section aims to assist beginners select the ideal package or software that best suits their needs. The first subsection focuses on causal discovery tools, while the second presents tools that support functionalities for both parameter learning and structure learning for the Bayesian network framework. Finally, the last subsection discusses commercial software that offers additional features such as optimized user-interfaces and professional customer support. Note that while the previous section provided a comprehensive overview of available solutions, this section shortlists and discusses only those we consider most suitable for beginners. It is important to note that while all the tools discussed in this section aim to uncover structure among variables, they differ in their underlying modeling assumptions and output types. Some tools (e.g., bnlearn, pgmpy, pyAgrum) are focused on Bayesian networks and provide probabilistic modeling capabilities, including structure and parameter learning as well as inference. Others (e.g., LiNGAM, CDT, causal-learn) are specialized for causal discovery and do not build a full probabilistic graphical model. Instead, these methods aim to recover a causal DAG under specific assumptions (e.g., linearity, non-Gaussianity, no hidden confounding). While the outputs may look similar (DAGs), their interpretation and use cases are different. We highlight these distinctions throughout the section to help readers select the tool that best fits their goals.

### 3.1 Tools for causal discovery (structure only, No probabilistic modeling)

When the goal is to discover the underlying structure among variables typically interpreted causally under certain assumptions without the need for full probabilistic modeling or inference, gCastle, CDT, and LiNGAM are three tools that represent viable solutions and provide easy access to those functionalities. In particular, gCastle by Huawei Noah’s Ark Lab is in our opinion one of the most accessible and comprehensive causal discovery open-source Python libraries at the time of writing this document. It offers various cutting-edge approaches for recovering the structure of causal networks ranging from score-based to gradient-based and hybrid algorithms. For each algorithm, the documentation offers a detailed practical example, making the tool very friendly to beginners. Various examples can also be found in Causal Inference and Discovery in Python (Part 3: Causal Discovery) (Molak, 2023), which offers the user the ability to dive deeper into any particular functionality offered by the tool. Moreover, gCastle can also be used via a GUI, which provides a friendlier version of the interface that does not involve coding. CDT is another great package that we feel confident in recommending. Its documentation contains several examples that will guide users step-by-step into their first structural learning attempts. CDT has the largest collection of algorithms for causal discovery among all the other reviewed tools for beginners, some of which can be run using Pytorch as well.

For time series data, the Longitudinal LiNGAM model (Kadowaki et al., 2013) extends the original LiNGAM framework (Shimizu et al., 2006) to account for temporal dynamics. It assumes that each variable is a linear function of its own past values and the past values of other variables, across a fixed number of time lags. The model assumes that the noise terms are continuous, non-Gaussian, and independent over time. These non-Gaussianity and independence assumptions are essential for identifying the direction of causal relationships from observational data, which would otherwise be unidentifiable under Gaussian noise.

The LiNGAM Python package includes implementations for various LiNGAM-based models, including the VAR-LiNGAM (vector autoregressive) model for time series. It offers theoretical background and practical examples for each model, making it a useful tool for both research and applied causal analysis.

When the goal is performing causal discovery on big data, Causal-Command represents a valid option. This Java library implements several algorithms for causal discovery and can be used via a shell script or as part of a Java-based application. We perceive this library to be less user-friendly compared to the ones mentioned above; thus, we deem Causal-Command a good fit for more intermediate or advanced users.

To conclude our assessment of tools specialized in causal structural learning, we consider CDT to be the best choice when having a large set of available methodologies is desirable. For example, CDT could be the most useful for training or educational purposes, where assessing and comparing the effectiveness of various methods is needed. CDT is also the best choice when an interface with Pytorch is required or preferred. While CDT offers a wide range of causal discovery algorithms, gCastle stands out for its user-friendly, code-free interface and well-curated documentation, making it especially accessible to non-programmers. Although both CDT and gCastle support linear non-Gaussian models, LiNGAM remains the most suitable tool when working specifically with this model type, as it is built for such scenarios.

### 3.2 Tools for Bayesian Networks (structure and parameter learning)

In many cases, one may wish to learn both the structure and the parameters of a probabilistic model using the Bayesian network framework. To this end, several tools extensively cover both functional areas while offering great simplicity of use. One of the most complete and well-maintained tools to date is bnlearn. Apart from the remarkable availability of built-in methods for parameter learning, structural learning, inference, missing data handling, and model validation strategies, what makes bnlearn stand out is its documentation and practical examples. Remarkably, most methods and examples are thoroughly explained in the books Bayesian Networks in R and Bayesian Networks With Examples in R (Scutari and Denis, 2021), of which the creator of bnlearn is co-author.

A valid alternative to bnlearn is represented by pgmpy. Unlike bnlearn, which provides methods for the static scenario only, pgmpy partially covers the dynamic case as well. This is an important feature, given that a great part of real-world problems and systems include time-dependent components. On the other hand, the range of algorithms available in pgmpy is more limited than in bnlearn, particularly for structure learning tasks. Nonetheless, pgmpy compensates for this limitation by offering more comprehensive documentation. Abundant examples are available in the practical notebooks section, along with tutorial notebooks, both of which are beneficial for taking the first steps into this field.

Another alternative to bnlearn is pyAgrum. Just like pgmpy, pyAgrum provides methods for static and dynamic scenarios, making it a valid option for time-dependent real-world problems. pyAgrum offers comprehensive documentation including tutorials, examples and applications with interactive widgets. An important resource offered by pyAgrum is a list of implemented solutions to the problems presented in the ‘Book of Why’ by Judea Pearl. PyAgrum not only provides rich and well-organized documentation, but also offers a wide array of structure learning methodologies. For example, it implements greedy hill climbing (GHC), local search with tabu-list (LS-TL), Multivariate Information-based Inductive Causation (MIIC), Chow-Liu, NB, TAN, and K2 algorithms.

A less sophisticated yet relevant package is the Python version of the original bnlearn (which is an R package). Although it is not as rich in methodologies as Pgmpy and the original bnlearn (only a handful of causal discovery algorithms are available in it), the Python version of bnlearn offers an intuitive interface and its documentation is as rich and well-curated as the original R version. The documentation not only presents many code snippets followed by the associated output but also provides a brief introduction to the theory behind it.

In conclusion, for those who are familiar with R, bnlearn represents the best choice, especially when coupled with the aforementioned books. For practitioners who prefer Python and/or need to model dynamic systems, pgmpy and pyAgrum are the best alternatives to bnlearn; the multitude of examples contained in pgmpy and pyAgrum documentation provides tremendous added value for beginners and/or practitioners moving their first steps in this field. The Python version of bnlearn offers a more straightforward interface than the other options; however, it does come with a limited number of structure learning algorithms, making it suitable for readers seeking to begin with simpler implementations.

### 3.3 Commercial software

For a wider and more flexible application of BNs frameworks in industry settings where cloud computing might be involved, the resulting models often need to be shared and accessed from a variety of devices, including mobile devices, where no-code solutions may be preferable. In addition, in these kind of scenarios, professional support is usually needed, making the open-source packages described in the previous sections unsuitable. In this section, we illustrate some practical commercial solutions that might satisfy the needs of larger industry organizations.

For this purpose, Bayes Server would be our recommended choice. A demo is available on their official website. The platform provides comprehensive documentation, including examples demonstrating how to interact with the graphical user interface. The documentation also features a code section that serves as a central repository of practical examples for working with the Bayes Server API. Additionally, the site showcases numerous real-world use cases across various domains, including aerospace and healthcare. Bayes Server is available under both commercial and academic licenses.

GeNIe by BayesFusion LLC is a valid alternative to Bayes Server. GeNIe makes use of the SMILE engine, a library of C++ classes that implement causal and parameter learning, as well as inference, which can be called via API. SMILE can be used via Java, Python, R, and. NET using the following wrappers: jSMILE (Java and environments that can instantiate and use the JVM), PySMILE (Python 2.7 and 3. x), rSMILE (R 3. x), SMILE.NET (.NET). Another component of GeNIe is BayesBox, an interactive repository where graphical models can be uploaded, shared, and consulted from a variety of devices, including mobiles.

A demo of BayesBox is available on the BayesFusion website. BayesFusion also provides detailed documentation, which includes information about GeNIe and its main features, as well as examples and introductory materials for SMILE. The support forum is also well-populated and can be a valuable resource for users.

A viable alternative to BayesServer and GeNIe is BayesiaLab. BayesiaLab has a commercial license and offers an intuitive GUI, APIs, and many useful resources, such as an ebook that includes several tutorials. Webinars, tutorials, and use cases that will help users navigate the multitude of features offered by BayesiaLab are also available. It is worthwhile to mention that the BayesiaLab API framework can be accessed using Java only.

In conclusion, both BayesServer and GeNIe can suit the aforementioned contexts. They are both equipped with a web platform that features a user-friendly interface and ready-to-use examples, and both software can be used on mobile devices. For BayesServer and GeNIe, pricing and licensing models can be the deciding factors in determining which tool best suits the reader’s needs after having tried their trial and demo versions. This might not apply to BayesiaLab, as users cannot try the software on the website before purchasing it. Additionally, BayesiaLab can only be used with Java.

## 4 Conclusion

This paper provides an overview of recent tools and software packages for Bayesian network structure and parameter learning, as well as methods specifically developed for causal discovery. The tools were reviewed from the perspective of a beginner seeking to gain hands-on experience in the field, and subjective recommendations were given about which tools are deemed more suitable. At the same time, it is important to acknowledge that the current landscape of BN tools remains fragmented. This fragmentation is largely due to the diverse range of assumptions, data types (e.g., discrete, continuous, mixed), and application domains (e.g., bioinformatics, social sciences, engineering) that BN modeling encompasses. As a result, many packages have been developed to cater to specific niches, leading to limited interoperability and a lack of standardization. Despite this, we believe the field is approaching a turning point. As methodologies that go beyond prediction are needed in real-world applications, there will be increasing pressure to integrate the software presented in this paper into more unified and user-friendly frameworks. Just as libraries like scikit-learn (Pedregosa et al., 2011) helped consolidate various machine learning algorithms into a common interface, we foresee the potential emergence of standardized libraries for BN modeling that balance flexibility with usability. Such developments would not only streamline experimentation and benchmarking but also lower the barrier of entry for practitioners and researchers across disciplines. Given the rapid evolution of this research field, updated versions of this document might be released periodically. The authors emphasize that all software contributions to this research field are instrumental in scientific advancement and complement each other in a beneficial way.

## Footnotes

1
https://bd2kccd.github.io/docs/causal-cmd/

2
https://erdogant.github.io/bnlearn/pages/html/index.html

3
https://www.bayesfusion.com/

4
https://www.bayesia.com/bayesia/bayesialab/bayesialab

5
https://www.bayesserver.com/
