# Correction: A guide to bayesian networks software for structure and parameter learning, with a focus on causal discovery tools

**DOI:** 10.3389/fsysb.2025.1717030

**Published:** 2025-10-23

**Authors:** Francesco Canonaco, Joverlyn Gaudillo, Nicole Astrologo, Fabio Stella, Enzo Acerbi

**Affiliations:** ^1^ Minutia.AI Pte. Ltd., Singapore, Singapore; ^2^ Department of Informatics, Systems and Communication, University of Milano-Bicocca, Milano, Italy

**Keywords:** structure learning, parameter learning, causal discovery algorithms, causal discovery, bayesian networks (BNs)

The **conflict of interest** statement was erroneously given as “Authors FC, JG, NA, and EA were employed by Minutia.AI Pte. Ltd.” The correct conflict of interest statement is “Authors FC, JG and NA were employed by Minutia.AI Pte. Ltd. EA has equity and is an advisor to Minutia.AI Pte. Ltd.”

The original article has been updated.

