# Supplementary material for: A guide to bayesian networks software for structure and parameter learning, with a focus on causal discovery tools
Source: Front Syst Biol. 2025 Aug 25;5:1631901. doi: 10.3389/fsysb.2025.1631901 (PMC12415694; doi:10.3389/fsysb.2025.1631901)
Supplement: Supplementary file 1 [file DataSheet1.pdf]

Supplementary Material

1 SUPPLEMENTARY TABLE

Table S1. List of available tools for Bayesian Networks.

Tool	Type[a]	Structure Learn[b]	Param. Learn[c]	Input Data[d]	Infer.[e]	Missing Data[f]	Constraint-Based Algo.[g]	Search/ Hybrid Algo.[h]	Scoring Fx[i]	Param. Learn Algo.[j]	GUI[k]	Lang.	Lic.[l]	OS[m]	API[n]	Last Up.	Doc.[o]
Bayes Server	S<br>D	Y	Y	C<br>D<br>M	Y	Y	PC	SS<br>H<br>CL<br>Clust<br>TAN	LL<br>BIC	MLE	Y	C#<br>Java<br>Python<br>R<br>Matlab	C	O<br>W<br>L	Y	2025	Home
Bayes-Fusion	S<br>D	Y	Y	C<br>D<br>H	Y	N	PC	ANB<br>GTT<br>BS<br>TAN	AIC<br>B<br>E<br>MDL<br>BDeu	EM<br>MLE<br>MAP	Y	C++	B	W	Y	2025	Doc
Bayesia-Lab	S<br>D	Y	Y	C<br>D<br>M	Y	Y	-	MWST<br>TS<br>EQ<br>SopLEQ<br>TO	MDL	MLE	Y	C++	C	O<br>W<br>L	Y	2023	Doc
blip	S	Y	Y	D	N	Y	-	WINA-SOBS<br>kMax<br>SEM-kMax	BIC<br>BDeu	MLE<br>BMA	N	Java<br>R	F	O<br>W<br>L	Y	2019	Rep
BNC-Weka	S	N	Y	D	N	N	ICS	HC<br>TAN<br>TS<br>RepHC<br>SA<br>Gen.S	AIC<br>BDe<br>MDL<br>E	MLE<br>BMA	Y	Java	F	O<br>W<br>L	Y	2022	Doc

Tool	Type[a]	Structure Learn[b]	Param. Learn[c]	Input Data[d]	Infer.[e]	Missing Data[f]	Constraint-Based Algo.[g]	Search/ Hybrid Algo.[h]	Scoring Fx[i]	Param. Learn Algo.[j]	GUI[k]	Lang.	Lic.[l]	OS[m]	API[n]	Last Up.	Doc.[o]
bnlearn (Python)	S	Y	Y	D	Y	Y	PC	TAN<br>HC<br>CL<br>Ex.S	BIC<br>K2<br>BDeu<br>BDs	MLE<br>BPE	N	Python	F	O<br>W<br>L	N	2022	Doc
bnlearn (R)	S	Y	Y	C<br>D<br>H	Y	Y	PC-Stable<br>GS<br>IAMB<br>FIAMB<br>In-IAMB<br>IAMB-FDR<br>SIHI-TONPC<br>MPC	HC<br>ARA-CNE<br>TAN<br>TS<br>CL<br>MMHC<br>HPC<br>RS-MAX2	LL<br>PLL<br>K2<br>AIC<br>BIC<br>BDeu<br>BDs<br>BDJ<br>BDIa<br>fNML<br>qNML<br>BGe	MLE<br>BPE<br>HEM	N	R	F	O<br>W<br>L	N	2025	Home
bnstruct	S	Y	Y	C<br>D	Y	Y	-	MMHC<br>MPC<br>HC<br>SEM<br>SMCS	AIC<br>BIC<br>BDeu	-	N	R	F	O<br>W<br>L	N	2024	Home

Tool	Type[a]	Structure Learn[b]	Param. Learn[c]	Input Data[d]	Infer.[e]	Missing Data[f]	Constraint-Based Algo.[g]	Search/ Hybrid Algo.[h]	Scoring Fx[i]	Param. Learn Algo.[j]	GUI[k]	Lang.	Lic.[l]	OS[m]	API[n]	Last Up.	Doc.[o]
causal-learn	S	Y	N	-	N	Y	PC<br>FCI<br>CDNOD	GES<br>Ex.S	BIC<br>BDeu	-	N	Python	F	O<br>W<br>L	N	2022	Doc
CDT	S	Y	N	C<br>D<br>H	N	N	FIAMB<br>GS<br>IAMB<br>In-IAMB<br>MPC<br>PC	ANM<br>BFit<br>CAM<br>CCDr<br>CDS<br>CGNN<br>GIES<br>GNN<br>GES<br>IGCI<br>Jarfo<br>LiN-GAM<br>NCC<br>RCC<br>RECI<br>SAM<br>SAMv1	BDe<br>BIC<br>GL-PIS<br>GL-POS<br>K2<br>MDL<br>SEM-GAM<br>SEM-LIN<br>BDs	-	N	Python	F	O<br>W<br>L	N	2023	Doc
CTBNLab	CT	Y	Y	-	N	N	CTPC<br>MBC-TPC	HC<br>RSHC<br>TS	BDe<br>CLL<br>LL	MLE<br>BPE	Y	Java	F	O<br>W<br>L	N	2022	Rep

Tool	Type[a]	Structure Learn[b]	Param. Learn[c]	Input Data[d]	Infer.[e]	Missing Data[f]	Constraint-Based Algo.[g]	Search/ Hybrid Algo.[h]	Scoring Fx[i]	Param. Learn Algo.[j]	GUI[k]	Lang.	Lic.[l]	OS[m]	API[n]	Last Up.	Doc.[o]
DEAL	S	Y	Y	C<br>D	N	N	-	GES	BDe<br>BF	BPE<br>MPP	Y	R	F	O<br>W<br>L	N	2018	Rep
gCastle	S	Y	N	C<br>D	N	N	PC	ANM<br>CORL<br>GNN<br>DLi-<br>NGAM<br>GAE<br>GOLEM<br>GES<br>HPCI<br>ICA-<br>LiNGAM<br>MCSL<br>NOTEARS<br>NOTEARS-<br>MLP<br>NOTEARS-<br>SOB<br>NOTEARS-<br>IOWRANK<br>PNL<br>RL<br>TTPM	LL<br>AIC<br>BIC<br>LSL	-	N	Python	F	O<br>W<br>L	N	2022	Rep

Tool	Type[a]	Structure Learn[b]	Param. Learn[c]	Input Data[d]	Infer.[e]	Missing Data[f]	Constraint-Based Algo.[g]	Search/ Hybrid Algo.[h]	Scoring Fx[i]	Param. Learn Algo.[j]	GUI[k]	Lang.	Lic.[l]	OS[m]	API[n]	Last Up.	Doc.[o]
GOB-NILP	S	Y	N	D	N	N	-	ILP	BDeu BGe	-	N	C++	F	O W	Y	2018	Home
Hugin expert	S D	Y	Y	C D M	Y	Y	NPC PC	TAN CL RP	AIC BIC	MLE	Y	Java	B	O W L	Y	2019	Home
LiN-GAM	S	Y	N	C	N	N	-	BUP-LiNGAM CAMUV DLi-NGAM LiM LiNA Long-LiNGAM MGD-LiNGAM RCD MGRCD RESIT VAR-LiNGAM VARMA-LiNGAM	-	-	N	Python	F	O W L	Y	2023	Doc
Open-Markov	S	Y	Y	D	Y	Y	PC	HC	K2	-	Y	Java	F	W	Y	2024	Home

Tool	Type[a]	Structure Learn[b]	Param. Learn[c]	Input Data[d]	Infer.[e]	Missing Data[f]	Constraint-Based Algo.[g]	Search/ Hybrid Algo.[h]	Scoring Fx[i]	Param. Learn Algo.[j]	GUI[k]	Lang.	Lic.[l]	OS[m]	API[n]	Last Up.	Doc.[o]
pcalg	S	Y	N	C<br>D<br>H	N	N	FCI<br>FCIJCI<br>AFCI<br>AAFCI<br>FCI+<br>PC<br>CPC<br>PCS<br>RFCI	ARGES<br>GIES<br>GES<br>LiNGAM<br>SMCS	-	-	N	R	F	O<br>W<br>L	N	2022	Doc
Pgmpy	S<br>D	Y	Y	C<br>D	Y	Y	PC	GES<br>HC<br>TrS<br>E<br>MMHC<br>Ex.S	BIC<br>K2<br>BDeu	EM<br>MLE<br>BPE<br>SEM	N	Python	F	O<br>W<br>L	N	2022	Doc
Pome-granate	S	Y	Y	C<br>D<br>M	Y	Y	-	HC<br>CL<br>EA*<br>ExSh	-	EM<br>MLE	N	Cython	F	O<br>W<br>L	Y	2025	Doc
py-Agrum	S<br>D<br>CT	Y	Y	C<br>D<br>M	Y	Y	MIIC	GHC<br>LSTL<br>K2<br>CL<br>NB<br>TAN	AIC<br>BD<br>BDeu<br>BIC<br>K2<br>LL	EM	N	C++<br>Python	F	O<br>W<br>L	N	2023	Home

Tool	Type[a]	Structure Learn[b]	Param. Learn[c]	Input Data[d]	Infer.[e]	Missing Data[f]	Constraint-Based Algo.[g]	Search/ Hybrid Algo.[h]	Scoring Fx[i]	Param. Learn Algo.[j]	GUI[k]	Lang.	Lic.[l]	OS[m]	API[n]	Last Up.	Doc.[o]
Tetrad	S	Y	Y	C<br>D<br>M	N	Y	PC<br>CPC<br>PCMax<br>FCI<br>FCI-Max<br>CFCI<br>RFCI<br>FCIIOD<br>Svar-FCI<br>Svar-GFCI<br>FAS<br>FASK<br>FASK-Vote<br>MGM<br>CCD	FGES<br>IMaGES<br>IMaGES-BOSS<br>FGES-FCI<br>GRaSP-FCI<br>BOSS-FCI<br>SPFCI<br>FGESMB<br>BOSS<br>BPC-MIMBuild<br>FOFC-MIMBuild<br>FTFC<br>SP<br>GRaSP<br>LVLite<br>DAGMA<br>ICA-LiNGAM<br>ICA-LiNGD<br>DLiNGAM<br>BOSS-LiNGAM<br>CStaR<br>OR	BDeu<br>MSep<br>BIC<br>SEM-BIC<br>EBIC<br>GIC<br>MVP<br>PP<br>ZSB<br>CG-BIC	MLE	Y	Java<br>Python<br>R	F	O<br>W<br>L	Y	2025	Doc

Tool	Type[a]	Structure Learn[b]	Param. Learn[c]	Input Data[d]	Infer.[e]	Missing Data[f]	Constraint-Based Algo.[g]	Search/ Hybrid Algo.[h]	Scoring Fx[i]	Param. Learn Algo.[j]	GUI[k]	Lang.	Lic.[l]	OS[m]	API[n]	Last Up.	Doc.[o]
UnB-Bayes	S	Y	Y	C D	Y	Y	-	-	B CBLA CBLB K2	-	Y	Java	F	O W L	N	2020	Home

LEGEND

[a] **Type:** Static (S), Dynamic (D), Continuous-Time (CT)

[b] **Structure Learn:** Yes (Y), No (N)

[c] **Param. Learn:** Yes (Y), No (N)

[d] **Input Data:** Continuous (C), Discrete (D), Mixed (M), Hybrid (H)

[e] **Infer.:** Yes (Y), No (N)

[f] **Missing Data:** Yes (Y), No (N)

[g] **Constraint-Based Algos:** Adaptative Anytime FCI (AAFCI), Anytime FCI (AFCI), Conservative Fast Causal Inference Algorithm (CFCI), Conservative Peter-Clark (CPC), Continuous-Time Peter-Clark (CTPC), Cyclic Causal Discovery (CCD), Expert in the Loop (E), Fast Adjacency Search (FAS), Fast Adjacency Search K (FASK), Fast Adjacency Search K Vote (FASKVote), Fast Causal Inference (FCI), Fast Causal Inference - Instrumental Observable Dependence (FCIIOD), Fast Causal Inference - Joint Causal Inference (FCIJCI), Fast Causal Inference Max (FCIMax), Fast Incremental Association Markov Blanket (FIAMB), Greedy Thick Thinning (GTT), Grow-Shrink (GS), Incremental Association Markov Blanket (IAMB), Incremental Association Markov Blanket - False Discovery Rate (IAMBFDR), Incremental Association Markov Blanket with Peter-Clark correction (IAMBnPC), Inductive Causation (IC), Integer Linear Programming (ILP), Interleaved Incremental Association Markov Blanket (InIAMB), Iterative Conditional Selection (ICS), Markov Blanket-based Continuous-Time Peter-Clark (MBCTPC), Max-Min Parents and Children (MPC), Mixed Graphical Model (MGM), Multivariate Information-based Inductive Causation (MIIC), Non-Parametric Conditional Independence Test (NPC), PC Select (PC simple) (PSC), Peter-Clark Algorithm (PC), Peter-Clark Max Algorithm (PCMax), Peter-Clark Stable Algorithm (PCStable), Really Fast Causal Inference Algorithm (RFCI), Semi-Interleaved HITON-Peter-Clark (SIHITONPC), Structural Vector Autoregressive Fast Causal Inference (SvarFCI), Structural Vector Autoregressive Generalized Fast Causal Inference (SvarGFCI), Tree Search (TrS)

[h] **Search/Hybrid Algos:** Adaptive Ridge Greedy Equivalence Search (ARGES), Additive Noise Model (ANM), Algorithm for the Reconstruction of Accurate Cellular Networks (ARACNe), Augmented Naive Bayes (ANB), Bayesian Fast Causal Inference (BFCI), Bayesian Optimization for Structure Search (BOSS), Bayesian Optimization for Structure Search - Fast Causal Inference (BOSSFCI), Bayesian Optimization for Structure Search - Linear Non-Gaussian Acyclic Model (BOSSLiNGAM), Bayesian Predictive Causal - Mutual Information Maximization Build (BPCMIMBuild), Bayesian Search (BS), Bivariate Fit (BFit), Bottom-Up Partitioned Causal Learning Linear Non-Gaussian Acyclic Model (BUPLiNGAM), Causal Additive Model (CAM), Causal Additive Models with Unobserved Variables (CAMUV), Causal Discovery in the presence of Nonstationary and/or Hetergeneous Data (CDNOD), Causal Discovery with Soft Interventions (CDS), Causal Generative Neural Networks (CGNN), Causal Order Recovery by Learning (CORL), Causal Structure and Representation Learning (CStaR), Chow-Liu (CL), Clustering (Clust), Complete Search (CS), Cyclic Causal Discovery with Regularization (CCDr), Direct Linear Non-Gaussian Acyclic Model (DLiNGAM), Directed Acyclic Graph Learning via Marginal Independence (DAGMA), Directed Acyclic Graph Learning with Graph Neural Networks (GNN), Exact A* (EA*), Exact Search (Ex.S), Exact Shortest (ExSh), Factorial Optimization for Feature Clustering - Mutual Information Maximization Build (FOFCMIMBuild), Fast Greedy Equivalence Search (FGES), Fast Greedy Equivalence Search - Fast Causal Inference (FGESFCI), Fast Greedy Equivalence Search - Markov Blanket (FGESMB), Find Two Factor Clusters (FTFC), First version of Structure Agnostic Model (SAMv1), Generalized Independence Noise condition-based Method (GIN), Gradient-based Score Propagation (GRaSP), Gradient-based Score Propagation Fast Causal Inference (GRaSPFCI), Graph Auto-Encoder (GAE), Graph Neural Networks for Structure Learning (GNN), Graph Optimization-based Learning of Equation Models (GOLEM), Greedy Hill Climbing (GHC), Greedy Interventional Equivalence Search (GIES), Greedy Search (GES), Hawkes Process-based Conditional Independence (HPCI) (HPCI), Hierarchical (H), Hill Climbing (HC), Hybrid Parents and Children (HPC), Independent Component Analysis for Linear Non-Gaussian Acyclic Model (ICALiNGAM), Independent Component Analysis for Linear Non-Gaussian Discovery (ICALiNGD), Independent Multiple-Gaussian Equivalent Search (IMaGES), Independent Multiple-Gaussian Equivalent Search with Bayesian Optimization Structure Search (IMaGESBOSS), Inferential Methods for Graph Estimation and Search (IMGES), Information-Geometric Causal Inference (IGCI), Integer Linear Programming (ILP), Joint Approximate Regression Feature Optimization (Jarfo), k-MAX (kMAX), Latent Variable Lite (LVLite), Linear Granger Causality (LGC), Linear Mixed (LiM), Linear Non-Gaussian Acyclic Model (LiNGAM), Linear Non-Gaussian Models for Latent Factors (LiNA), Local Search with Tabu-List (LSTL), Longitudinal LiNGAM (LongLiNGAM), Low-Rank Approximation for Non-Equivalent Transformations for Additive Noise Models (NOTEARSlOWRANK), Max-Min Hill Climbing (MMHC), Monte Carlo Structure Learning (MCSL), Multi-Group Direct LiNGAM (MGDLiNGAM), Multi-Group Repetitive Causal Discovery (MultiGroupRCD), MultiGroup Repetitive Causal Discovery (MGRCD), Naive Bayes (NB), Neural Causal Criterion (NCC), Non-Equivalent Transformations for Additive Noise Models (NOTEARS), Non-Equivalent Transformations for Additive Noise Models with Multi-Layer Perceptron (NOTEARSMLP), Non-Equivalent Transformations for Additive Noise Models with Structural Optimization Bias (NOTEARSSOB), Orientation Algorithms (R3, RSkew, Skew) (OR), Probabilistic Nonlinear Learning (PNL), Random-restart HC (RSHC), Rebane-Pearl (RP), Regression Error-based Causal Inference (RECI), Regression with Subsequent Independence Test (RESIT), Regression-based Causal Criterion (RCC), Reinforcement Learning for Causal Discovery (RL), Repeated Hill Climbing (RepHC), Repetitive Causal Discovery (RCD), Restricted Structural Maximum Algorithm 2 (RSMAX2), Search and Score (SS), Silander-Myllymaki Complete Search (SMCS), Sparse Variational Graphical Fast Causal Inference (SvarGFCI), Sparsest Permutation (SP), Sparsest Permutation - Fast Causal Inference (SPFCI), Structural Equation Modeling (SEM), Structural Expectation Maximization with k-MAX (SEMkMAX), Structure Agnostic Model (SAM), Tabu Search (TS), Tree-augmented Naive Bayes (TAN), Tree-Thresholded Pairwise Mutual Information (TTPM), Vector Autoregressive Models - Linear Non-Gaussian Acyclic Model (VARLiNGAM), Vector Autoregressive Moving Average - Linear Non-Gaussian Acyclic Model (VARMALiNGAM), Weighted Incremental Association-based Structure Obtention System (WINASOBS)

[i] **Scoring Fx:** Akaike Information Criterion (AIC), Bayes Factor (BF), Bayesian Dirichlet (BD), Bayesian Dirichlet Equivalent (BDe), Bayesian Dirichlet Equivalent Uniform (BDeu), Bayesian Dirichlet Sparse (BDs), Bayesian Gaussian Equivalent (BGe), Bayesian Information Criterion (BIC), Bayesian Score (B), Bayesian-Dirichlet equivalent uniform with an imaginary sample size "a" (BDIa), Bayesian-Dirichlet with Jeffrey's Prior (BDJ), Causal Bayesian Learning - Score A (CBLA), Causal Bayesian Learning - Score B (CBLB), Conditional Gaussian Bayesian Information Criterion (CGBIC), Conditional Log-Likelihood (CLL), Directional Separation (DSeparation), Entropy (Entropy), Entropy (E), Extended Bayesian Information Criterion (EBIC), Factorized Normalized Maximum Likelihood (fNML), Gaussian L0-penalized Intervention Score (GLPIS), Gaussian L0-penalized Observational Score (GLPOS), Generalized Information Criterion (GIC), Generalized Score with Cross Validation (GSCV), Generalized Score with Marginal Likelihood (GSMI), K2 (K2), L0 Norm Penalty (L0NP), Log-Likelihood (LL), M-Separation (MSep), Minimum Description Length (MDL), Mixed Variable Polynomial (MVP), Poisson Prior (PP), Predictive Log-Likelihood (PLL), quasi Normalized Maximum Likelihood (qNML), Structural Equation Modeling - Generalized Additive Model (SEMGAM), Structural Equation Modeling - Linear Model (SEMLIN), Structural Equation Modeling Bayesian Information Criterion (SEMBIC), Zhang-Shen Bound (ZSB)

[j] **Param. Learn Algos:** Bayesian Model Averaging (BMA), Bayesian Parameter Estimation (BPE), Exhaustive Search (Exh.S), Expectation Maximization (EM), Genetic Search (Gen.S), Hierarchical and Expectation Maximization (HEM), Master Prior Procedure (MPP), Maximum A Posterior (MAP), Maximum Likelihood Estimation (MLE), Simulated Annealing (SA), Structural Equation Model Estimators (SEM)

[k] **GUI:** Yes (Y), No (N)

[l] **Lic. (License):** Free (F), Commercial (C), Both (B)

[m] **OS (Operating System):** OSX (O), Windows (W), Linux (L)

[n] **API:** Yes (Y), No (N)

[o] **Doc. (Documentation):** Doc (Documentation), Rep (Repository), Home (Homepage)
